# Treatment of Full-Thickness Rotator Cuff Tendon Tear Using Umbilical Cord Blood-Derived Mesenchymal Stem Cells and Polydeoxyribonucleotides in a Rabbit Model

**DOI:** 10.1155/2018/7146384

**Published:** 2018-05-15

**Authors:** Dong Rak Kwon, Gi-Young Park, Sang Chul Lee

**Affiliations:** ^1^Department of Rehabilitation Medicine, Catholic University of Daegu School of Medicine, Daegu, Republic of Korea; ^2^Department and Research Institute of Rehabilitation Medicine, Yonsei University College of Medicine, Seoul, Republic of Korea

## Abstract

**Objective:**

The aim of this study was to investigate regenerative effects of ultrasound- (US-) guided injection with human umbilical cord blood-derived mesenchymal stem cells (UCB-MSCs) and/or polydeoxyribonucleotide (PDRN) injection in a chronic traumatic full-thickness rotator cuff tendon tear (FTRCTT) in a rabbit model.

**Methods:**

Rabbits (*n* = 32) were allocated into 4 groups. After a 5 mm sized FTRCTT just proximal to the insertion site on the subscapularis tendon was created by excision, the wound was immediately covered by a silicone tube to prevent natural healing. After 6 weeks, 4 injectants (0.2 mL normal saline, G1-SAL; 0.2 mL PDRN, G2-PDRN; 0.2 mL UCB-MSCs, G3-MSC; and 0.2 mL UCB-MSCs with 0.2 ml PDRN, G4-MSC + PDRN) were injected into the FTRCTT under US guidance. We evaluated gross morphologic changes on all rabbits after sacrifice. Masson's trichrome, anti-type 1 collagen antibody, bromodeoxyuridine, proliferating cell nuclear antigen, vascular endothelial growth factor, and platelet endothelial cell adhesion molecule stain were performed to evaluate histological changes. Motion analysis was also performed.

**Results:**

The gross morphologic mean tendon tear size in G3-MSC and G4-MSC + PDRN was significantly smaller than that in G1-SAL and G2-PDRN (*p* < 0.05). However, there were no significant differences in the tendon tear size between G3-MSC and G4-MSC + PDRN. In G4-MSC + PDRN, newly regenerated collagen type 1 fibers, proliferating cell activity, angiogenesis, walking distance, fast walking time, and mean walking speed were greater than those in the other three groups on histological examination and motion analysis.

**Conclusions:**

Coinjection of UCB-MSCs and PDRN was more effective than UCB-MSC injection alone in histological and motion analysis in a rabbit model of chronic traumatic FTRCTT. However, there was no significant difference in gross morphologic change of tendon tear between UCB-MSCs with/without PDRN injection. The results of this study regarding the combination of UCB-MSCs and PDRN are worth additional investigations.

## 1. Introduction

Rotator cuff tendon tears (RCTTs) are the most common tendon injury in adults and affect about 30% of people over 60 years of age [1]. Although surgical repair of a RCTT is one of the most common orthopedic procedures, the failure rate for rotator cuff tendon repair ranges widely from 20% to 90% [2]. The current therapeutic approaches do not achieve physiological restoration of the RCTT, and the quality of the repaired tendon is not optimal [3]. These deficiencies have driven attempts to regenerate RCTT with the use of biological adjuvants. Mesenchymal stem cells (MSCs) have been proposed as an attractive alternative to overcome the limitations of the current treatments [4]. Of the various MSCs, human umbilical cord blood-derived MSCs (UCB-MSC) have a greater therapeutic potential than MSCs derived from other tissues because of attributes that include the ability to home in on injured tissue, low immunogenicity, multidirectional differentiation, and extensive secretion profiles [5]. The function of autologous MSCs in patients with advanced age or significant comorbidities is impaired, and allogeneic UCB-MSCs may therefore be of particular benefit in the elderly or those with multiple comorbidities [6]. In addition, UCB-MSCs can be produced commercially in larger quantities with the same quality.

Polydeoxyribonucleotide (PDRN) is a biological adjuvant which has the same advantages as UCB-MSCs in terms of commercial mass production. PDRN is a mixture of DNA polymers featuring a chain with length ranging from 50 to 2000 bp. It is extracted from the trout sperm and purified as a preparation containing a high percentage of DNA. PDRN is a source of pyrimidines and purines and stimulates nucleic acid synthesis through the salvage pathway [7]. PDRN can induce angiogenesis and collagen synthesis and also has an anti-inflammatory activity [8]. One recently published study reported the effectiveness of PDRN in the treatment of chronic rotator cuff disease [9].

The aim of our study was to evaluate the efficacy of UCB-MSCs and/or PDRN injections in regenerating RCT in a rabbit model. This is the first report to compare the regenerative effects of stem cell and PDRN in a model of RCTT.

## 2. Material and Methods

### 2.1. Animal Model

Twelve-week-old, male, New Zealand white rabbits (*n* = 32) were housed in separate metal cages at a temperature of 23 ± 2°C and a relative humidity of 45 ± 10%. They had free access to tap water and were fed a commercial rabbit diet. None of the rabbits received additional exercise, and all were allowed to do normal activities in a 65 × 45 × 30 cm cage. Animal experiments were performed in accordance with internationally accredited guidelines and approved by the University of School of Medicine Animal Care and Use Committee.

Anesthesia was induced using isoflurane (JW Pharmaceutical, Goyang, South Korea) vaporized in oxygen and delivered using a large animal cycling system. Under general anesthesia, 5 mm diameter full-thickness RCTTs (FTRCTTs) were created just proximal to the insertion site on the left subscapularis tendon by punch biopsy excision using a Biopsy Punch 5 mm LZ (SFM, Wächtersbach, Germany). Each excision wound was immediately covered with a resorbable round silicone Penrose drainage tube (Sewoon Medical Co. Ltd., Cheonan, South Korea) to induce a chronic rotator cuff tear. The incision was closed using subcutaneous and skin sutures [10].

### 2.2. Mesenchymal Stem Cells

UCB was collected from the umbilical veins of pregnant women after neonatal delivery with informed consent. MSCs were isolated from the UCB and cultivated [11, 12]. The cells expressed cluster of differentiation 105 (CD105, 96.93%), CD90 (98.96%), CD29 (98.26%), CD166 (81.29%), CD73 (83.49%), CD45 (0.26%), CD14 (1.0%), and human leukocyte antigen D related (HLA-DR, 0.18%). They also expressed pluripotent markers including octamer-binding transcription factor 4 (30.5%) and stage-specific embryonic antigen 4 (67.7%). UCB-MSCs can differentiate into cell types including respiratory epithelium, osteoblasts, chondrocytes, and adipocytes with specific in vitro induction stimuli [13–15]. We confirmed the differentiation potential and karyotypic stability of the UCB-MSCs up to passage 11. UCB-MSCs were mixed with viscous hyaluronic acid.

### 2.3. Animal Grouping and Injection

**Figure 1:**
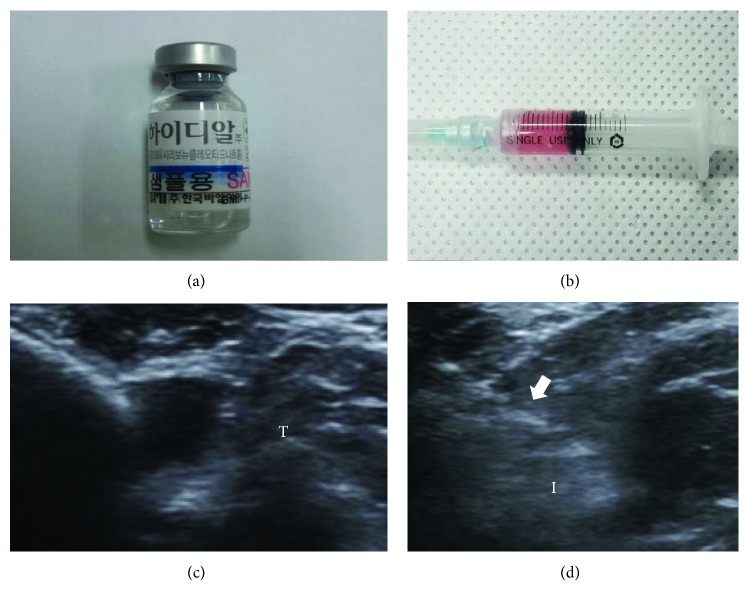
Human umbilical cord blood-derived mesenchymal stem cells (MSCs), polydeoxyribonucleotide (PDRN), and ultrasound images. (a) PDRN. (b) MSCs. (c) Injection was made in the left shoulder subscapularis full-thickness tears under ultrasound guidance. (d) Longitudinal ultrasound image shows the needle (arrow) in the left shoulder subscapularis of the rabbit. T: tendon; I: injectant.

**Figure 2:**
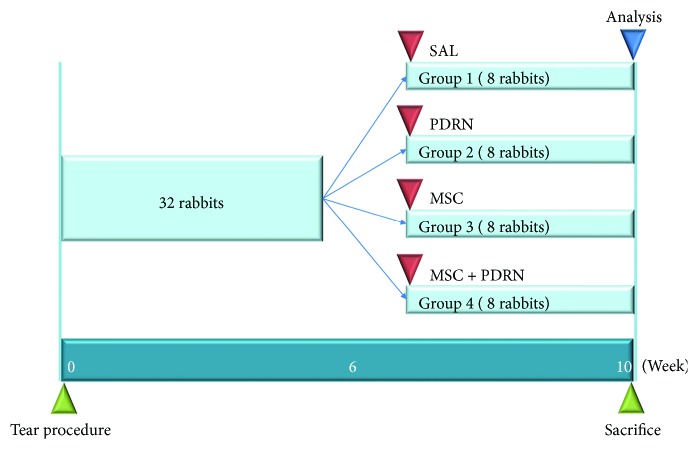
Timeline of saline, PDRN, MSC, and PDRN with MSC injection. Normal saline (0.2 mL; group 1: SAL), PDRN (0.2 mL; group 2: PDRN), MSC (0.2 mL; group 3: MSC), and MSCs with PDRN (both 0.2 mL; group 4: MSC + PDRN) were injected under ultrasound guidance into the left shoulder subscapularis full-thickness tears 6 weeks after the tears were created. The analysis including gross morphology of tear site, histologic examination, and motion analysis was performed 4 weeks after injection of four different solutions. All rabbits were euthanized by carbon monoxide inhalation 4 weeks after injection of the different solutions. PDRN: polydeoxyribonucleotide; MSC: human umbilical cord blood-derived mesenchymal stem cell.

Six weeks after the excisions, the inserted tubes were removed to induce chronic FTRCTT. The site of each full-thickness subscapularis tendon tear was confirmed, and the incision was using subcutaneous and skin sutures. Rabbits were randomly allocated into four treatment groups (*n* = 8 per group) 6 weeks after excision. Group 1 (G1-SAL) was injected with 0.2 mL of normal saline, group 2 (G2-PDRN) with 0.2 mL commercially obtained PDRN (Hidr, BMI Korea, Seoul, Korea; Figure 1(a)), group 3 (G3-MSC) with 0.2 mL (1 × 106 cells) UCB-MSCs (Figures 1(b)–1(d)), and group 4 (G4-MSC + PDRN) with 0.2 mL UCB-MSCs and 0.2 mL PDRN. All rabbits were euthanized 4 weeks postinjection (Figure 2). All injections were performed under ultrasound (US) guidance using an US system with an 18~5 MHz multifrequency linear transducer (EPIQ 5; Philips Healthcare, Andover, MA, USA). No medication was administered, and all rabbits were immobilized in the equinus position using an elastic bandage for 2 days after the injection.

### 2.4. Gross Morphology Examination

Gross morphologic examinations were conducted after each rabbit was euthanized. Each tendon tear was classified as partial or full thickness. Gross morphologic tendon tears were photographed to the subscapularis tendon tear using a clear plastic ruler near the center of the tear site to permit calculation of size using ImageJ software (National Institute of Health, Bethesda, MD) by tracing the outlined tear edge preinjection and at 4 weeks postinjection.

### 2.5. Histological Examination Tissue Preparation

The rabbits were sacrificed under general anesthesia after all intramuscular injections. The tear area of the subscapularis tendon was segmented and fixed with neutral-buffered formalin for 24 hours. Each specimen was embedded in paraffin (Paraplast; Oxford, St. Louis, MO, USA) and sliced sagittally into 5 *μ*m thick serial sections. The specimens were stained with hematoxylin-eosin (H-E) and Masson's Trichrome (MT) stains and examined by light microscopy.

### 2.6. Immunohistochemistry

Immunohistochemical staining of tendon sections was done for collagen fibers using mouse anti-collagen 1 monoclonal antibody (COL-1, Abcam, Cambridge, UK) and for the marker of proliferating cells using 5-bromo-2′-deoxyuridine (BrdU; Cayman Chemical, Ann Arbor, MI, USA). To accomplish BrdU staining, rabbits were postoperatively given 25 mg/kg BrdU intraperitoneally. Twenty-four hours later, each rabbit was sacrificed and paraffin-embedded sections were prepared. The sections were incubated in 0.1% trypsin for 10 minutes at 37°C and 1 N HCl for 30 minutes at 56°C to denature the DNA. Endogenous peroxidases were inhibited by preincubation in 0.3% hydrogen peroxide (H_2_O_2_) in phosphate-buffered saline (PBS) for 30 minutes, and nonspecific protein binding was blocked in PBS containing 10% normal horse serum (Vector Laboratories, Burlingame, CA, USA) for 30 minutes. The sections were incubated in monoclonal anti-BrdU (1 : 100, Sigma-Aldrich, St. Louis, MO, USA) for 2 hours at room temperature and washed three times with PBS. The secondary antibody (1 : 100), biotinylated anti-mouse IgG (Vector Laboratories), was placed on the sections for 1 hour at room temperature, followed by three washes with PBS. Avidin-biotin-peroxidase complex (ABC, Vector Laboratories) was placed on the sections for 1 hour, followed by three PBS washes, and further followed by a peroxidase reaction using 0.05 M Tris-HCl (pH 7.6) containing 0.01% H_2_O_2_ and 0.05% 3,3′-diaminobenzidine (Sigma-Aldrich). The sections were counterstained with hematoxylin and then mounted. The slides were examined with Axiophot Photomicroscope (Carl Zeiss, Germany) and AxioCam MRc5 (Carl Zeiss, Germany). The number of immunopositive cells or nuclei was counted.

The tendon sections were stained for the marker of proliferating cells using mouse anti-proliferating cell nuclear antigen monoclonal antibody (PCNA, PC10, Santa Cruz Biotechnology, Santa Cruz, CA, USA), angiogenic markers using anti-vascular endothelial cell growth factor polyclonal antibody (VEGF, A-20, Santa Cruz Biotechnology), and anti-platelet endothelial cell adhesion molecule-1 polyclonal antibody (PECAM-1, M-20, Santa Cruz Biotechnology). Sections were immunostained for the marker of type I and type III collagen fibers using either mouse COL-I or mouse COL III. The paraffin-embedded sections were cleared, dehydrated, and washed with PBS. Antigen retrieval was performed using ethylenediaminetetraacetic acid (EDTA) buffer (1 mM EDTA, pH 8.0) for 30 minutes at 95°C followed by cooling. Endogenous peroxidases were inhibited by preincubation in 0.3% H_2_O_2_ in PBS for 30 minutes, and nonspecific protein binding was blocked in PBS containing 10% normal horse serum for 30 minutes. The sections were incubated in primary antibodies (1 : 100 ~ 1 : 200) for 2 hours at room temperature and washed three times with PBS. The secondary antibody (1 : 100), biotinylated anti-mouse IgG or biotinylated anti-rabbit IgG or biotinylated anti-goat IgG (Vector Laboratories), was placed on the sections for 1 hour at room temperature and washed three times with PBS. Sections were exposed to ABC for 1 hour, washed three times with PBS, and subjected to a peroxidase reaction using 0.05 M Tris-HCl (pH 7.6) containing 0.01% H_2_O_2_ and 0.05% 3,3′-diaminobenzidine (DAB, Sigma-Aldrich). The sections were counterstained with hematoxylin and then mounted. The slides were examined using an Axiophot Photomicroscope equipped with an AxioCam MRc5. Each slide was evaluated according to the intensity of positive immunostaining.

Thirty randomly selected fields from each group were photographed using the AxioCam MRc5 interfaced with the Axiophot Photomicroscope. The AxioVision SE64 (Carl Zeiss, Germany) program was used for analysis. A semiquantitative scoring system for the nuclear BrdU, PCNA, and cytoplasmic markers (VEGF, and PECAM-1) was used considering the staining intensity and extent of the area; this approach is widely accepted and has been used in previous studies [16, 17]. Briefly, the proportion of positive stained cells was scored as 0 (no cells stained positive), 1 (1%–10% stain-positive cells), 2 (11%–33% stain-positive cells), 3 (34%–66% stain-positive cells), and 4 (67%–100% stain-positive cells). The intensity of COL I or COL III positive immunostaining was graded as −, +, ++, and +++ (negative, slight positive, moderate positive, and strong positive staining, resp.).

### 2.7. Motion Analysis

The motion analysis of the rabbits was conducted at preinjection and 4 weeks postinjection. Rabbits were habituated for 30 minutes to the open field before motion analysis was performed [18]. They were placed on a 3 × 3 M arena and allowed to freely explore the field for 5 minutes. Their movements were individually assessed using a video-tracking system equipped with a camera (Smart; Panlab, Barcelona, Spain) that recorded the rabbit's horizontal activity. Five-minute walking distance, fast walking time, and mean walking speed were measured.

### 2.8. Statistical Analysis

Statistical analyses were performed with the SPSS program for Windows program, version 19.0 (SPSS Inc., Chicago, IL, USA). In addition to standard descriptive statistical calculations (means and standard errors), ANOVA was used to determine statistical differences among intragroup and intergroup. When ANOVA yielded significant results indicating that the group was significantly different from the others, Tukey's test was also performed. The mean values were followed by 95% confidence intervals, and all the data are expressed as the means ± standard error. The statistically significant levels were predetermined at *P* < 0.05 and *P* < 0.001.

## 3. Results

**Figure 3:**
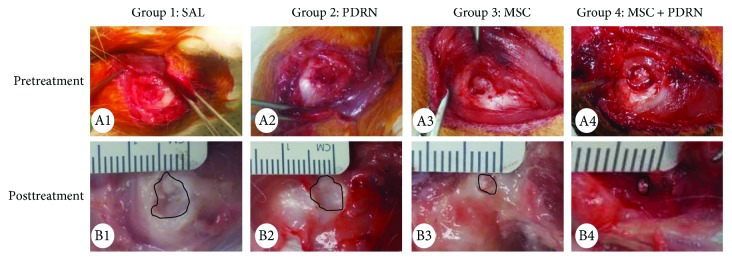
Gross morphological (A1–B4) findings of the subscapularis tendons in groups 1, 2, 3, and 4. (A1–A4) Pretreatment images. FTT is observed in all four groups. (B1–B4) Posttreatment images. FTT is shown and no gross morphologic changes between pretreatment and four weeks posttreatment in G1-SAL and G2-PDRN. There are significant differences in gross morphologic changes between pretreatment and four weeks posttreatment in G3-MSC and G4-MSC + PDRN. Normal saline (0.2 mL; group 1: SAL), PDRN (0.2 mL; group 2: PDRN), MSC (0.2 mL; group 3: MSC), and MSCs with PDRN (both 0.2 mL; group 4: MSC + PDRN). MSC: human umbilical cord blood-derived mesenchymal stem cell; PDRN: polydeoxyribonucleotide; FTT: full-thickness tendon tear.

**Figure 4:**
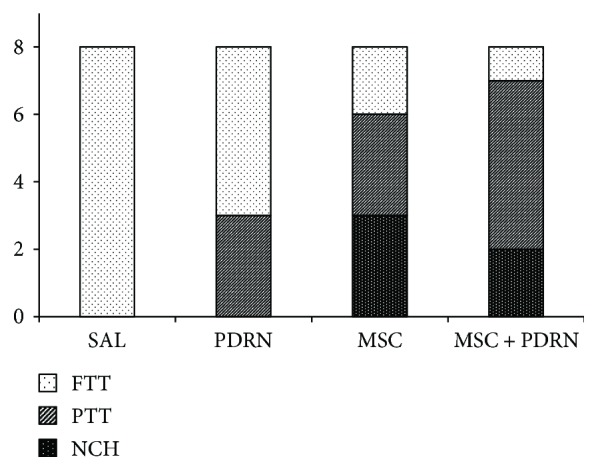
Gross morphology of tear site at 4 weeks postinjection. FTT was observed in all eight rabbits in G1-SAL. In G2-PDRN, a PTT was observed in three rabbits and FTT in five rabbits. In G3-MSC, a PTT was observed in three rabbits, FTT in two rabbits, and CH in three rabbits. In G4-MSC + PDRN, a PTT was observed in five rabbits, FTT in one rabbit, and CH in two rabbits. SAL (normal saline 0.2 mL); PDRN (PDRN 0.2 mL); MSC (MSC 0.2 mL); and MSC + PDRN (MSC 0.2 mL with PDRN 0.2 mL). PDRN: polydeoxyribonucleotide; MSC: human umbilical cord blood-derived mesenchymal stem cell; FTT: full-thickness tendon tear; PTT: partial-thickness tendon tear; CH: nearly complete healing.

At 4 weeks postinjection, RCTT was observed in all eight rabbits in G1-SAL. In G2-PDRN, a partial-thickness subscapularis tendon tear was observed in three rabbits (37.5%) and full-thickness tendon tear in five rabbits (62.5%). In G3-MSC, a partial-thickness subscapularis tendon tear was observed in three rabbits (37.5%), full-thickness tendon tear in two rabbits (25%), and nearly complete healing in three rabbits (37.5%). In G4-MSC + PDRN, a partial-thickness subscapularis tendon tear was observed in five rabbits (62.5%), full-thickness tendon tear in one rabbit (12.5%), and nearly complete healing in two rabbits (25%) (Figures 3 and 4). There were significant differences in gross morphologic changes between preinjection and four weeks postinjection in G3-MSC and G4-MSC + PDRN.

**Figure 5:**
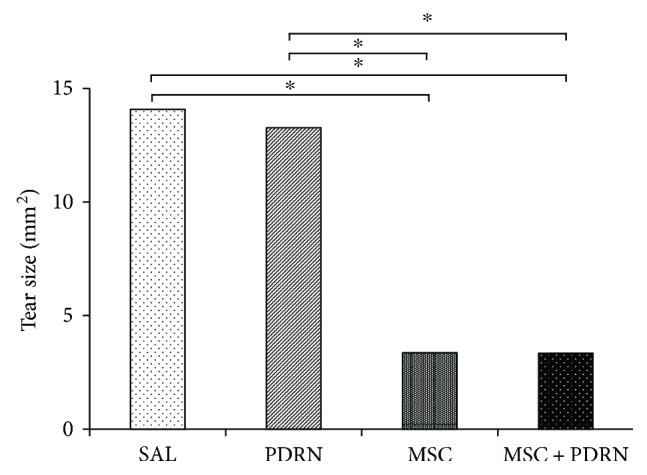
Subscapularis tendon tear size at 4 weeks postinjection. ^∗^*P* < 0.05, one-way ANOVA, Tukey's post hoc test between two groups. SAL (group 1, normal saline 0.2 mL); PDRN (group 2, PDRN 0.2 mL); MSC (group 3, MSC 0.2 mL); and MSC + PDRN (group 4, MSC 0.2 mL with PDRN 0.2 mL). PDRN: polydeoxyribonucleotide; MSC: human umbilical cord blood-derived mesenchymal stem cell.

The gross morphologic mean tendon tear size of each group at 4 weeks postinjection was 13.08 mm^2^ (G1-SAL), 13.27 mm^2^ (G2-PDRN), 3.36 mm^2^ (G3-MSC), and 3.35 mm^2^ (G4-MSC + PDRN). The size of the tear in G3-MSC and G4-MSC + PDRN was significantly smaller than that in G1-SAL and G2-PDRN (*P* < 0.05). There were no significant differences in gross morphologic changes and tendon tear size between G3-MSC and G4-MSC + PDRN (Figures 3 and 5).

**Figure 6:**
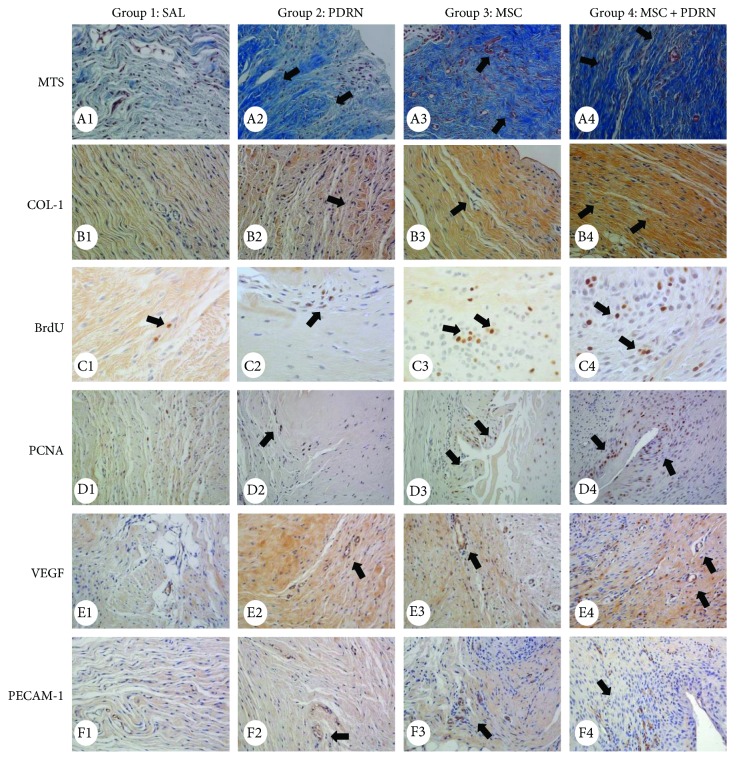
Histological findings of the subscapularis tendons in group 1 (SAL), group 2 (PDRN), group 3 (MSC), and group 4 (MSC + PDRN). (A1–A4) Newly regenerated tendons are shown in the blue-stained fibers (black arrow; Masson's trichrome stain; X200) in groups 2, 3, and 4. Few regenerative collagen fibers were seen in group 1. (B1–B4) Regenerated tendon fibers (black arrow; X200) were stained with anti-type 1 collagen antibody in groups 2, 3, and 4. Few regenerated tendon fibers were seen in group 1. (C1–D4) Numerous BrdU- and PCNA-stained cells (black arrow, X400, X200) were observed in regenerated tendon fibers in groups 2, 3, and 4. Few BrdU- and PCNA-stained cells were observed in group 1. (E1-F4) Numerous VEGF-positive cells and PECAM-1 positive microvascular densities (black arrows, X200) were observed in groups 2, 3, and 4. Few VEGF-positive cells and PECAM-1-positive microvascular densities were observed in group 1. MTS: Masson's trichrome stain; COL-1: collagen type 1; BrdU: 5-bromo-2′-deoxyuridine; PCNA: proliferating cell nuclear antigen; MSC: human umbilical cord blood-derived mesenchymal stem cell; PDRN: polydeoxyribonucleotide; VEGF: vascular endothelial growth factor; PECAM: platelet endothelial cell adhesion molecule.

**Figure 7:**
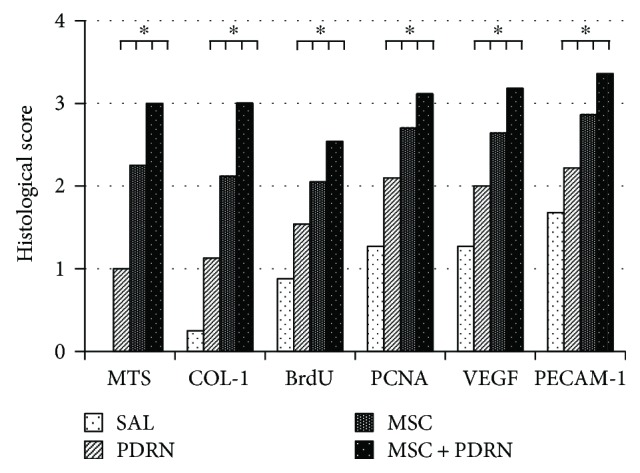
Semiquantitative score of histological findings and immunoreactivity of stain. The immunoreactivity of MTS and anti-type 1 collagen antibody stain and proportion of BrdU-, PCNA-, VEGF-, and PECAM-1-positive cells were scored as detailed in Materials and Methods. ^∗^*P* < 0.05 one-way ANOVA, Tukey's post hoc test among groups. SAL (group 1, normal saline 0.2 mL); PDRN (group 2, PDRN 0.2 mL); MSC (group 3, MSC 0.2 mL); and MSC+PDRN (group 4, MSC 0.2 mL with PDRN 0.2 mL). MSC: human umbilical cord blood-derived mesenchymal stem cell; PDRN: polydeoxyribonucleotide; VEGF: vascular endothelial growth factor; PECAM: platelet endothelial cell adhesion molecule.

**Figure 8:**
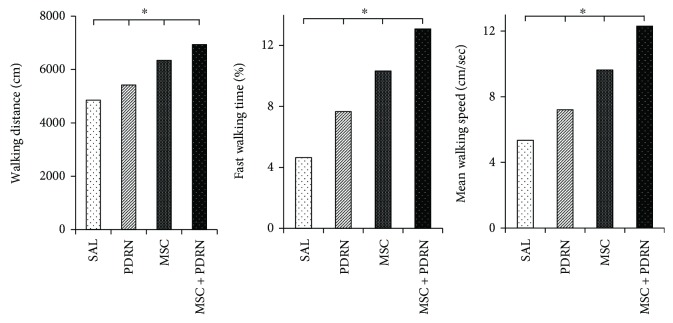
Motion analysis of the rabbits at 4 weeks postinjection. In G4-MSC + PDRN, walking distance, fast walking time, and mean walking speed are greater than those in the other three groups on motion analysis. ^∗^*P* < 0.05 one-way ANOVA, Tukey's post hoc test among groups. Group 1 (normal saline 0.2 mL). Group 2 (PDRN 0.2 mL). Group 3 (MSC 0.2 mL). Group 4 (MSC 0.2 mL with PDRN 0.2 mL). MSC: human umbilical cord blood-derived mesenchymal stem cell; PDRN: polydeoxyribonucleotide.

On H-E staining, a parallel arrangement of hypercellular fibroblastic bundles was observed in G1-SAL, G2-PDRN, and G3-MSC. This arrangement was hardly observed in G1-SAL. On MT staining, regenerated collagen fibers were observed and were stained with COL-1 in G2-PDRN, G3-MSC, and G4-MSC + PDRN, but staining was rare in G1-SAL (Figure 6A1–B4). Numerous PCNA and BrdU-stained cells were also observed in regenerated collagen fibers in G2-PDRN, G3-MSC, and G4-MSC + PDRN, but only rarely in G1-SAL (Figure 6C1–D4). Immunohistochemistry staining revealed numerous VEGF-positive cells, and PECAM-1 positive microvascular densities were observed in G2-PDRN, G3-MSC, and G4-MSC + PDRN (Figure 6E1–F4). In G4-MSC + PDRN, newly regenerated collagen type 1 fibers, cell proliferation, angiogenesis, walking distance, fast walking time, and mean walking speed were greater than those in the other three groups on histological examination and motion analysis (Figures 7 and 8, Table 1).

## 4. Discussion

The major finding of this study is the superior therapeutic effect of coinjection with UCB-MSCs together with PDRN, compared with UCB-MSCs alone when evaluated functionally and histologically. PDRN has regenerative properties and stimulates wound healing by enhancing angiogenesis and production of VEGF through the adenosine A_2_ receptor stimulation [19]. To our knowledge, this is the first study to analyze the difference between stem cells and PDRN or combination therapies for the regeneration of the rotator cuff tear. UCB-MSCs and PDRN can be produced in large quantities while maintaining the same quality. It would be ideal if the combination of PDRN and UCB-MSCs was synergistic in terms of their effects or if PDRN was effective enough in the regeneration of RCTTs to replace expensive stem cell-based therapy.

PDRN has been shown to improve the skin repair process and enhance wound breaking strength in diabetic animals. These effects were associated with a marked increase in the expression of VEGF, a master regulator of angiogenesis that is impaired in diabetes-related wound disorders [7]. We postulate that VEGF is a major growth factor that accelerates the healing process by stimulating new vessel formation in regions of poor circulation including full-thickness tendon tear.

UCB-MSCs are an attractive stem cell source. Advantages include noninvasive collection, superior tropism, and differentiation potential [20]. In addition, UCB-MSCs are less immunogenic; a recent study that used UCB-MSCs in anterior cruciate ligament reconstruction in a rabbit model showed no evidence of immune rejection [21]. The present findings are consistent with this benign immune behavior.

PDRN contains a mixture of deoxyribonucleotide polymers with chain lengths ranging from 50 to 2000 bp and is a source of purine and pyrimidine deoxynucleosides/deoxyribonucleotides and bases [22]. PDRN has regenerative properties and stimulates wound healing by enhancing angiogenesis and production of VEGF through the adenosine A_2_ receptor stimulation [19]. PDRN has no antigenic properties since it consists of low-molecular weight DNA fractions that can be defined as deoxyribonucleotide linear polymers [23]. Animal studies have demonstrated that PDRN is not lethal and is nontoxic to the liver, lungs, brain, skeletal muscle, and heart [24].

Another potential advantage of the combination of PDRN and UCB-MSCs is that the effect of exogenous MSCs is not dose-dependent. If the effect of exogenous MSCs is not proportional to dose, it is better to combine PDRN than to administer large amounts of MSCs to regenerate RCTT. A recent study in a porcine model of chronic myocardial infarction found that cardiosphere-derived cells at escalating doses led to equally enhanced preservation of cardiac function and tissue remodeling without a dose-efficacy relationship [25]. Our previous (unpublished) findings agree with this finding. We previously demonstrated that the injection of UCB-MSCs under US guidance has a regenerative effect for chronic full-thickness RCTTs. However, there were no differences in these regenerative effects between the high and the low doses of the UCB-MSCs, which mean that the benefits of UCB-MSCs were not dose-dependent in our rabbit model. Although further studies are needed, the fact that the effect of UCB-MSCs is not dose-dependent makes the rational combination with PDRN more compelling.

In addition to comparing the effect of UCB-MSC and PDRN on FTRCTT, the current study has important features in the method. The first feature is the use of musculoskeletal US for US-guided injection and evaluation of RCTT size after injection. We tried to validate the effect of only UCB-MSCs or PDRN injection, not an adjuvant therapy for surgical treatment in the FTRCTT model. The effect of MSCs combined with surgical repair has been reported in RCTT. However, combined with surgical repair, MSCs are an adjuvant therapy, and the effect of surgical repair overshadows their exact role in regenerating torn tendons [26]. In this context, musculoskeletal US can become a critical interventional tool for regenerative injection therapies because US-guided injections allow the stem cells to be selectively administered to the target area [18, 27]. Musculoskeletal US can also allow the relatively accurate assessment of the extent of the tear during the follow-up period before sacrificing the rabbit. We confirmed full-thickness tendon tear by US 6 weeks after establishing the rotator cuff tear. The four different injectants were separately introduced into the tear area under US guidance. The size of the tendon tear was measured by US at preinjection and 4 weeks postinjection. The motion analysis of the rabbits was also done to evaluate the improvement in functional ability rather than the mechanical properties of a regenerated tendon [18]. Motion analysis including walking distance, fast walking time, and mean walking speed for 5 minutes was conducted at preinjection and 4 weeks postinjection. This analysis is not yet proven to be superior to the mechanical testing that is frequently used in animal models of the rotator cuff tear [28–30]. However, motion analysis is a potentially important tool to assess the therapeutic effect of the rotator cuff tear, since functional tests have been demonstrated to be an important tool to assess the effectiveness of treatments for FTRCTT in human studies [31].

Chronic RCTTs adversely affect and hamper the surgical repair of lesions [32]. In particular, massive FTRCTTs are usually associated with myotendinous retraction, atrophy, and fatty infiltration of the muscles, which are bad prognostic factors for surgical outcomes. Thus, a “chronic” FTRCTT model is needed to accurately assess the clinical utility of MSCs in humans. For this study, we used a rabbit model of a chronic traumatic RCTT after 6 weeks of trauma. Studies in the rabbit supraspinatus muscle have shown fatty degeneration beginning as early as 4 weeks, with a peak at 6 weeks and slow reversal by 12 weeks [33]. FTRCTT becomes irreparable after approximately 6 weeks as the result of excessive tendon retraction and muscle atrophy and stiffening [34]. Accordingly, we selected 6 weeks for the chronic injury although we did not confirm whether the “chronic” findings were present in the tendon injuries [33–35].

There were significant differences in gross morphologic changes between preinjection and 4 weeks postinjection in groups 3 and 4, but not in G1-SAL and G2-PDRN. The difference between the pairs of groups concerns the presence or not of UCB-MSCs. G2-PDRN rabbits treated with PDRN did not show the regeneration effect. This proves that the therapeutic effect does not result from PDRN alone. Although the initial focus of MSC treatment of musculoskeletal injuries was based on their ability to differentiate into several cell types, recent studies suggest that the beneficial effect of stem cell-based therapy depends mainly on a paracrine effect [25, 36]. Paracrine signaling from MSCs modulates many cellular responses including survival, proliferation, migration, and gene expression [37, 38]. We observed hypercellular fibroblastic bundles composed of type I collagens in the regenerated tendon 4 weeks after the injection of UCB-MSCs. The main cause of unsuccessful tendon tear surgery is the formation of a fibrovascular scar enriched in type III collagens, which are mechanically weaker than type I, the main component of the extracellular matrices in tendons [39, 40]. The observation supports the idea that the injection of USB-MSCs promotes the normal tendon healing process in the repair of chronic RCTTs, even though USB-MSCs did not presently differentiate into the target cell type.

The gross morphologic mean tendon tear size of each group at 4 weeks postinjection were 13.08 mm^2^ (G1-SAL), 13.27 mm^2^ (G2-PDRN), 3.36 mm^2^ (G3-MSC), and 3.35 mm^2^ (G4-MSC + PDRN). Since there were no significant differences in gross morphologic changes and tendon tear size between G3-MSC and G4-MSC + PDRN, we were unable to demonstrate whether the combined therapy with PDRN and USB-MSCs was more effective than USB-MSCs alone. However, in G4-MSC + PDRN, newly regenerated collagen type 1 fibers, cell proliferation, angiogenesis, walking distance, fast walking time, and mean walking speed were greater than those in the other three groups based on the histological and motion analyses.

Therefore, when combined with USB-MSCs, PDRN might have a synergic or additive effect in the treatment of RCTTs.

The exact mechanism of treatment for both USB-MSCs and PDRN remains unknown. Paradoxically, the value of the combined effect of PDRN and MSCs is due to the limitations of the MSC regenerative effect. Accumulating evidence suggests that the regenerative properties of exogenous MSCs are mainly due to the paracrine mechanism because the engrafted MSCs have poor differentiation and survival rates [41–43]. This paracrine action may be accounted for, at least in part, by microvesicles (MVs) released from MSCs, which deliver proteins, bioactive lipids, and nucleic acids to injured cells [44]. Although exogenous MSCs can be considered a potential therapeutic tool and can contribute to tissue repair, the extent of improvement of injured tissues has not been correlated with cellular engraftment and differentiation of MSCs to tissue cells. It is necessary to develop strategies to obtain sufficient amounts of MVs. Since the paracrine effect of MSCs may not be proportional to the amount of MSCs administered, other additional sources are needed to fully provide the necessary protein (or its precursors), bioactive factors, and nucleic acids [19, 22, 23]. It is assumed that the PDRN can fulfill this role.

In the current study, 0.2 mL of PDRN with or without UCB-MSCs was injected into the right FTSSCT under US guidance. This dose volume of PDRN was the same dose as the stem cells. The optimal dose or route of administration of PDRN required for regenerating the rotator cuff was not established. We estimated and used 0.2 mL of PDRN because rabbits weigh about 5% of the weight of a typical adult. One study used 3 mL in patients with chronic rotator cuff tendinopathy [9]. However, in studies on human subjects, PDRN was injected directly into lesions to demonstrate therapeutic efficacy. A half vial of PDRN (1.5 mL) was injected each week for 3 weeks for the treatment of plantar fasciitis [8], and 5.625 mg in 3 mL of PDRN was injected at weekly intervals for 3 weeks to treat chronic rotator cuff tendinopathy [9]. To confirm the regenerative effect of the rotator cuff tear of the PDRN, it is necessary to clarify the optimal dose and route of administration of PDRN.

There are some limitations in our study. First, we created 5 mm diameter FTRCTTs near the insertion site on the left subscapularis tendon by punch biopsy excision. After each excision was made, each wound was immediately covered with a round silicone tube to induce the chronic rotator cuff tear model. Each wound was closed using subcutaneous and skin sutures. However, these tears were in the tendon body, not exactly at the insertion site, which is not reestablished following surgical repair. This outcome is associated with high recurrence rates [45]. Second, complete regeneration did not occur. Current strategies for using stem cells to regenerate FTRCTTs can be combined with mechanical stimulation, the topography of the extracellular matrix, growth and differentiation factors, gene transfection, and coculture with tendon tissues or cells. Future studies are needed that use UCB-MSCs and/or PDRN with these factors [46]. Third, the optimal dose of UCB-MSCs was determined based on previous studies. However, as mentioned above, the dose of PDRN was determined to be the same as UCB-MSCs for comparison. However, this dose may not be the proper dose of PDRN. Fourth, we did not perform the biomechanical test of the regenerative tendon. Last, there would also have been more “complete” rotator cuff healing if outcomes were measured at 8 weeks or more instead of at 4 weeks.

## 5. Conclusion

There was no significant difference in gross morphologic change of the tendon tear between UCB-MSCs only and the combination with PDRN injection in a rabbit model of chronic traumatic FTRCTT. However, coinjection of UCB-MSCs and PDRN was more effective than the injection of UCB-MSCs alone in histological and motion analyses. These results of this study regarding the combination of UCB-MSCs and PDRN warrant more investigations.

## Figures and Tables

**Table 1:** Semiquantitative score of histological findings, immunoreactivity of stain, and motion analysis according to treatment groups at 4 weeks after injection.

	Groups (injection regimens)			
	0.2 mL NS (G1-SAL)	0.2 mL PDRN (G2-PDRN)	0.2 mL MSC (G3-MSC)	0.2 mL MSC + 0.2 mL PDRN (G4-MSC + PDRN)
*Histological score*				
MTS	0.0 ± 0.0^∗^	0.9 ± 0.56^∗^	2.2 ± 0.42^∗^	3.0 ± 0.0^∗^
Anti-type collagen 1	0.2 ± 0.42^∗^	1.1 ± 0.31^∗^	2.1 ± 0.31^∗^	3.0 ± 0.0^∗^
BrdU	0.88 ± 0.84^∗^	1.54 ± 1.13^∗^	2.05 ± 1.16^∗^	2.54 ± 1.11^∗^
PCNA	1.27 ± 0.91^∗^	2.10 ± 1.07^∗^	2.70 ± 1.08^∗^	3.11 ± 1.00^∗^
VEGF	1.27 ± 0.94^∗^	2.00 ± 1.07^∗^	2.64 ± 0.92^∗^	3.18 ± 0.79^∗^
PECAM-1	1.68 ± 0.93^∗^	2.22 ± 0.92^∗^	2.86 ± 0.98^∗^	3.36 ± 0.75^∗^
*Motion analysis*				
Walking distance (cm)	4728.37 ± 137.27^∗^	5416.62 ± 323.27^∗^	6343.62 ± 213.57^∗^	6932.37 ± 107.74^∗^
Fast walking time (%)	5.62 ± 1.42^∗^	8.32 ± 0.34^∗^	10.33 ± 2.48^∗^	12.07 ± 1.77^∗^
Mean walking speed (cm/sec)	6.3 ± 0.57^∗^	8.36 ± 0.39^∗^	9.62 ± 1.78^∗^	12.3 ± 1.13^∗^

Values are the mean ± SD. The immunoreactivity of MTS and anti-type 1 collagen antibody stain and proportion of BrdU-, PCNA-, VEGF-, and PECAM-1-positive cells were scored as detailed in Materials and Methods. NS: normal saline; MSC: human umbilical cord blood-derived mesenchymal stem cell; PDRN: polydeoxyribonucleotide; G1-SAL: 0.2 mL normal saline group; G2-PDRN: 0.2 mL PDRN; G3-MSC: 0.2 mL UCB-MSCs; G4-MSC + PDRN: 0.2 mL UCB-MSCs with 0.2 ml PDRN; MTS: Masson's trichrome stain; BrdU: bromodeoxyuridine; PCNA: proliferating cell nuclear antigen; VEGF: vascular endothelial growth factor; PECAM-1: platelet endothelial cell adhesion molecule. *P* < 0.05 one-way ANOVA, Tukey's post hoc test among groups.
